# The impact of off-pump surgery in end-organ function: practical end-points

**DOI:** 10.1186/s13019-015-0362-2

**Published:** 2015-11-10

**Authors:** Haralabos Parissis, Simon Mbarushimana, Bandigowdanapalya C. Ramesh, Mondrian Parissis, Savvas Lampridis, Peter Mhandu, Bassel Al-Alao

**Affiliations:** 1 Cardiothoracic Department, Royal Victoria Hospital, Grosvenor Road, Belfast, BT12 6BA Northern Ireland; 2 Cardiopulmonary Transplantation Department, Freeman Hospital, Newcastle Upon Tyne, NE7 7DN UK; 3 Thoracic Department, Mayo Clinic, 200 First Street SW, Rochester, MN 55905 USA

**Keywords:** Off-Pump coronary surgery, Coronary artery bypass surgery, Beating Heart surgery

## Abstract

Most surgeons perform coronary bypass surgery with the aid of cardiopulmonary bypass, which inflicts a massive systemic inflammatory response to the body leading to adverse clinical outcome. In an attempt to make CABG less invasive, interest have been diverted to the off pump technique.

The current review attempts to bring an insight onto the last ten years knowledge on the off-pump impact in end organ function, with an aim to draw some clear conclusions in order to allow practitioners to reflect on the subject.

## Background

On pump coronary artery surgery with the aid of cardiopulmonary bypass, in theory, inflicts a massive systemic inflammatory response resulting in altered microcirculation. Important features of this inflammatory reaction include the activation of complement and leukocytes, the release of proinflammatory cytokines, alterations in the metabolism of nitric oxide, and an increase in the production of oxygen-free radicals, which in some cases may lead to oxidant stress injury [1].

Several therapeutic measurements including the use of steroids, heparin-coated CPB circuits, and hemofiltration have been reported to reduce the inflammatory reaction induced by CPB and its consequences.

Furthermore, The use of Cardiopulmonary Bypass (CPB), together with bleeding (during and after surgery), frequent blood analyses (before, during and after surgery), hemodilution, significant shift of intravascular volume and mechanical trauma of blood cells, cause significant changes in the three major cellular components of the hematopoietic system [2].

Following red blood loss, anemia is associated with a range of postoperative consequences (stroke, acute myocardial infarction), major side effects, re-hospitalization, duration of stay in the Intensive Care Unit and hospital stay, mortality 30 days after the intervention [3].

Moreover CABG surgery is associated with an increase in inflammatory markers and serum M30 levels, indicating epithelial/endothelial apoptosis in the early post operative period [4].

A more radical and effective way of counteracting the effects of the inflammatory reaction and oxidative stress may be the omission of CPB itself.

Off pump coronary artery surgery, through obviating the use of artificial circuit was thought to reduce myocardial injury, reduce neurocognitive dysfunction and Cerebrovascular accident (CVA), reduce organ injuries, reduce incidence of atrial fibrillation, reduce systemic inflammatory response, reduce stress hormone response, reduce blood loss/transfusion requirement and last but not least, reduce cost. So, do we have evidence for these potential benefits of off-pump coronary surgery?

The trend for advocating off-pump surgery has fluctuated during the last decade and is now in steady decline. Currently, this technique is used in fewer than 1 in 5 patients who undergo surgical coronary revascularization [5].

In this review article we attempt to examine the impact of off-pump surgery at the tissue level.

### Myocardial injury

There is a strong association between elevation of Creatine Kinase MB (CK-MB) or troponin levels within the first 24 h post Coronary artery Bypass surgery and increased intermediate- and long-term risk of mortality [6].

It has to be said that a lower creatine kinase-MB threshold, i.e., ≥80 μg/L, had been chosen to define postoperative myocardial infarction by some institutions and this has an impact when various studies reporting outcomes.

A meta-analysis [7] to assess the accuracy of increased troponin concentrations for the prediction of mid-term (> or = 12 months) mortality after coronary artery bypass graft showed that there is an association between peri-operative troponin release and short-term all-cause mortality after adult cardiac surgery.

Contrary to some believe that off-pump surgery is protecting the myocardium from damage, Karu et al. [8] looked into Coronary sinus and arterial blood samples for measurement of troponin I, creatine kinase MB, lactate, glutathione, and interleukin-6 were taken from 23 patients during off pump surgery. Significant increases in interleukin-6 were found in coronary sinus samples after 5 and 20 min of reperfusion. The authors concluded that surgical trauma during off-pump coronary surgery is sufficient to activate an inflammatory response in the myocardium.

More specifically [9], in Off-pump surgery the post operative levels of cardiac troponin and creatine kinase –MB, influence survival rates at 3 years; by enlarge high perioperative levels of cardiac enzymes is associated with adverse outcomes.

During Off-pump beating heart surgery, by maintaining pulsatile flow and coronary perfusion, there is in theory, a constant source of myocardial blood flow and therefore better protection than on-pump coronary artery bypass grafting.

In a randomized-prospective design by Rastan et al., [10] 40 elective patients with normal EF and three vessels coronary artery disease were assigned to off pump or on pump surgery. Before coronary occlusion and 1, 30, 60, and 90 min after reperfusion with the LIMA graft, coronary sinus (CS) blood was sampled to determine intraoperative myocardial ischemia (pH, lactate, pO2) and oxidative stress (malondialdehyde, MDA). Additionally to CS blood arterial blood was analyzed 4, 12, and 24 h postoperatively to determine myocardial necrosis (CK-MB, cardiac troponin I), myocardial dysfunction (NT-proBNP) and inflammation (C-reactive protein). All the enzymes studied were higher post operatively on the on pump group. Therefore off pump revealed less myocardial injury than on pump.

In an interesting study by Chowdhury et al. [11] fifty patients were randomly assigned to on-pump or off-pump coronary artery bypass grafting. All cardiac biomarkers were measured in serial venous blood samples drawn before heparinization in both groups and after aortic unclamping at 1, 2, 4, 8, 24, 48, and 72 h in the on-pump group. In the off-pump group samples were taken after the last distal anastomosis and at same time intervals as in the on-pump group. The authors concluded that the total amount of heart-type fatty acid-binding protein, cardiac troponin I, and high-sensitivity C-reactive protein released was significantly higher in the on-pump group; and therefore Off-pump coronary artery bypass grafting provides better myocardial protection than on-pump coronary artery bypass grafting.

The concept of remote ischemic post-conditioning (RIPC) is that transient ischemia in one tissue protects distant tissues from sustained ischemia. In previous studies, RIPC was induced by inflating a blood pressure cuff, on an upper or lower limb, for certain amount of time.

An interesting RCT by Hong et al. [12] have shown that RIPC by lower limb ischemia was found to reduce myocardial enzyme elevation by almost half. The authors concluded that RIPC by lower limb ischemia decreased postoperative myocardial enzyme elevation by almost half postoperatively in patients undergoing off pump surgery.

Brain natriuretic peptide (BNP) has been recognized as a useful marker for acute and chronic left ventricular dysfunction. A study by Wang et al. [13] was designed to evaluate the clinical relevance of BNP before and after off-pump coronary artery bypass surgery. The authors concluded that baseline BNP level correlated with preoperative ventricular function and longer durations of ventilation and hospital stay after off pump surgery. BNP increased early after operation. However, postoperative BNP did not correlate with myocardial injury or clinical results after off pump surgery.

A very interesting study from Bristol, UK [14] attempt to explain the hypothesis that off pump surgery reduces the myocardial injury associated with CPB. They looked at gene expressions linked with inflammatory responses. The authors concluded that off-pump surgery is associated with fewer alterations in gene expression connected with inflammation, apoptosis, and remodeling seen after on-pump surgery with CPB and ischemic-cardioplegic arrest.

A prospective randomized trial by Serrano et al. [15] compare the inflammatory response and myocardial injury from patients submitted to Off pump CABG with those that undergo On pump CABG.

Postoperative CK-MB and cTnI levels were 13.9 +/− 6.5 IU and 19.0 +/− 9.0 ng/mL for Off pump CABG versus 29.5 +/− 11.0 IU and 31.5 +/− 10.1 ng/mL for On pump CABG (*P* < .01).

At 24 h, for Off pump CABG and On pump CABG: IL-6 was 37 +/− 38 and 42 +/− 41 g/mL; IL-8, 33 +/− 31 and 60 +/− 15 pg/mL; soluble P-selectin, 99 +/− 26 and 172 +/− 30 pg/mL; soluble ICAM-1, 227 +/− 47 and 236 +/− 87 ng/mL; and CRP, 10 +/− 11 and 14 +/− 13 mg/L (*P* < .01). From this important study it appears clearly that the absence of CPB during CABG preserves better the myocardium and attenuates inflammation.

Lastly, in a meta-analysis of observational studies reported by Attaran et al. [16] off pump versus on-pump revascularization was compared between female patients. Interestingly Peri-operative myocardial infarction rate was significantly lower with Off pump, however, this did not not translate into a reduction in 30-day mortality and Off pump did not significantly improve other cardiovascular, renal or neurological outcomes.

In summary, lot of studies suggested a biochemical attenuation of inflammatory response when CPB was avoided, however all studies failed to translate this finding into a significant clinical improvement.

### Lung injury

What is the role of off pump coronary surgery in reducing the incidence of pulmonary complications, following cardiac surgery?

In theory, cardiopulmonary bypass is believed to reduce postoperative lung function by promoting atelectasis, an increase in parenchymal lung water, and by inflammatory molecules and micro emboli damage to the lung architecture.

This injury leads mostly to a postoperative interstitial pulmonary oedema and abnormal gas exchange.

And although an attenuated inflammatory response has been shown following off-pump coronary artery bypass, the degree of postoperative lung dysfunction is similar with that of conventional Coronary Artery Bypass Grafting CABG [17].

Indeed, some clinical studies [18] showed that, both On-pump and Off-pump CABG patients experienced similar degrees of decreased PaO2 and increased P(A-a)O2, but a higher percentage of pulmonary shunt fraction after On-pump operations.

In a large cohort study by Reddy et al. [19], the use of cardiopulmonary bypass was an independent risk factor for prolonged intubation. However, when investigators looked at the levels of inflammatory mediators in bronco-alveolar lavage fluid, Tumor necrosis factor (TNF-alpha) and Interleukin (IL-8) were increased in both on and off-pump groups, similarly [20].

There are no randomized control trials to have assigned COPD patients to off versus on pump. Nevertheless, a prospective trial of 200 patients randomized to off pump versus on pump, examined preoperative and postoperative respiratory compliance, fluid balance, hemodynamic, arterial blood gases, chest radiographs, spirometry, pulmonary complications, and time to extubation [21]. Interestingly, intra-operative fluid intake was higher in the off pump group (4541 +/− 1311 mL vs. 3585 +/− 1033 mL, *p* < 0.0001) and possibly that resulted in higher postoperative pulmonary arterial diastolic pressure (15.0 +/− 5.5 mm Hg vs. 11.8 +/− 5.2 mm Hg, *p* < 0.0001) and central venous pressure. The reason for that was fluid resuscitation in order to maintain haemodynamic stability during grafting the lateral and inferior wall of the heart. As per authors, immediate postoperative PaO2 on fraction of inspired oxygen of 1.0 was higher after off pump and extubation was earlier. However this last statement could be biased due to various confounding factors.

Engels et al. [22] used serial measurements of plasma concentrations of Clara cell 16 kD (CC16) protein, Surfactant protein D (SP-D), Elastase and Myeloperoxidase on blood samples from 40 patients who underwent coronary artery bypass grafting with cardiopulmonary bypass (CABG, *n* = 20) or without cardiopulmonary bypass (Off pump, *n* = 20).

The increase of SP-D and CC16 between pre-operative concentrations and concentrations at the end of cardiopulmonary bypass, correlated with the Aa-O2 gradient at 1 h on the ICU.

Furthermore, SP-D and CC16 were higher in CABG than in off pump at the end of surgery Syed et al. [23] randomized 75 consecutive patients into off and on pump groups. Alveolar-arterial oxygen difference (A-aO(2) difference) was calculated pre-operatively, then 2 and 4 h post-operatively. PaO(2)/FiO(2) ratio and respiratory index (RI) were calculated 2 and 4 h post-operatively.

Alveolar-arterial O(2) gradient sharply increased in the immediate post-operative period, from 27 mmHg pre-operatively, to 227 mmHg 2 h post-operatively, then declined to 152 mmHg 4 h post-operatively. PaO(2)/FiO(2) ratio and RI also showed severe worsening 2 h post-operatively, with marked improvement at 4 h. The pattern of physiological deterioration of gas exchange was similar in both the groups.

In a retrospective study looking into the impact of off pump on patients with COPD, Kerendi and colleagues [24] concluded that after propensity score adjustment, off pump was associated with a significantly reduced incidence of prolonged ventilation, pneumonia, and intensive care unit stay. Looking carefully into the paper, the greatest impact of off pump in reducing prolonged ventilation, pneumonia, adult respiratory distress syndrome (ARDS), and mortality, was among those patients without preoperative lung disease. Unfortunately, in patients with COPD, comparison between the 2 techniques showed non-significant differences.

Another small prospective randomize trial [25] looked into spirometric evaluation, blood gas measurements and alveolo-arterial oxygen gradient, at the fourth and 10th postoperative days, in on pump versus off pump patients. The sample of 42 individuals recruited had no COPD. Both groups showed reduction in all values measured however according to the authors the off-pump patients showed significantly less reduction in values than the on-pump group.

In summary, at the present time, all the studies in the subject are small randomized trials that have shown a “biochemical trend” towards attenuation of lung injury when CPB is avoided, however this has failed to mark a clinical impact.

Up till today, there is no robust evidence to support that off pump should be performed preferentially among patients with preoperative lung disease. An RCT is needed to answer this dilemma.

### Renal injury

The ischemic heart disease population is ageing and suffers with co morbidities, including renal impairment. As a result of epidemiological studies [26] only 24.7 % of the patients undergoing CABG have normal glomerular filtration rate (GFR), 51 % have mild renal impairment, 20.1 % have moderate and 2.0 % have severe renal impairment; finally 2.2 % are on dialysis preoperatively. Hospital mortality according to this study was worse correlating with the levels of renal severity: mild renal impairment (odds ratio [OR] 1.42); moderate renal impairment (OR 3.55); severe renal impairment (OR 8.84); and dialysis-dependent (OR 9.64). Likewise long term survival was worse across the levels of renal dysfunction. I think that this underscores the fact that renal dysfunction is an adverse predictor overall and its impact on outcome is more profound that the type of procedure advocated (On pump versus Off Pump).

A meta-analysis of randomized and observational studies recruiting more than 27,000 patients [27] sought to examine the controversy as to whether an off-pump technique can reduce post CABG renal injury. Unfortunately, the majority of the studies were biased, by recruiting low risk patients. Nevertheless there were some important messages from this study:

1) There was a significant reduction in overall acute renal injury (odds ratio [OR], 0.57) and acute renal injury requiring renal replacement (OR, 0.55) in the off-pump group compared with the on pump group.

2) In Randomized Control Trials (RCTs), overall acute renal injury was significantly reduced in the off-pump group (OR, 0.27); however, no statistically significant difference was noted in acute renal injury requiring renal replacement (OR, 0.31).

3) In the observational cohort, both overall acute renal injury (OR, 0.61) and acute renal injury requiring renal replacement (OR, 0.54) were significantly less in the off-pump group.

Along the same lines a smaller meta-analysis [28] looking into 4819 patients with normal renal function preoperatively, showed that the incidence of acute renal injury in the on-pump CABG group was 4.0 %, and the incidence of new dialysis requirement 2.4 %. Off-pump CABG was associated with 40 % lower odds of postoperative acute renal injury (OR 0.60; 95 % CI 0.43, 0.84; *P* = 0.003) and non-significant 33 % lower odds for dialysis requirement (OR 0.67; 95 % CI 0.40, 1.12; *P* = 0.12).

When pre-existing non-dialysis dependent renal dysfunction is diagnosed, off-pump surgery provides better renal protection than the conventional on-pump technique, was reported in a small prospective randomized trial by Ooi et al. [29]. However, both groups returned to the preoperative values by 4 weeks.

In a cohort study looking into creatinine clearance values preoperatively and day 1, 2 and 4 postoperatively Abu-Omar et al. [30] have reported significantly lower creatinine clearance postoperatively in patients with diabetes (*P* < 0.001) and advanced age (*P* < 0.001). The on-pump group of 1145 patients had significantly lower postoperative creatinine clearance in comparison to the off-pump group (*P* = 0.01) of 435 patients. The effect remained consistent after adjusting for risk factors such as age, diabetes, gender, LV function and preoperative creatinine clearance.

Nigwekar et al. [31] conducted a meta analysis on the relationship of off pump surgery and acute kidney injury (AKI). When the authors analyzed the data from RCTs, they found that overall AKI was significantly reduced in the Off pump group; however, no statistically significant difference was noted in AKI requiring Renal replacement therapy (RRT). In the observational cohort, both overall AKI and AKI requiring RRT were significantly less in the OPCAB group. RCTs were noted to be underpowered and biased toward recruiting low-risk patients. In this important study the conclusion suggests a reduction in AKI using the Off pump technique;

Another recent [32], rather large meta analysis enrolled only RCTs. This paper summarizes very well the current knowledge on the subject: 33 RCTs with 17,322 patients were enrolled in our study. Patients in the off-pump CABG group had overall lower incidence of AKI (19.1 %) compared to the on-pump CABG group (22.2 %). There was a protective effect of off-pump CABG on the incidence of AKI compared to the on-pump CABG group. However, there was no significant difference in the need for dialysis in the off-pump group compared to the on-pump group and furthermore no benefits on survival among patients undergoing off-pump CABG.

### Gastrointestinal injury

Gastro intestinal (GI) complications by means of GI bleed, ileus, pancreatitis, ischemic bowel and cholecystitis, is relative uncommon in patients following CABG. Therefore, in order to identify differences between on versus off pump surgery in those patients a large-scale prospectively conducted study is required. This study is lacking from the literature up till now.

A small-scale retrospective study showed no significant difference in the total number of GI complications between the off-pump and on-pump groups [33]. Likewise, a retrospective report by Musleh et al. [34] showed that the incidence of GI complications was 1.2 % (*n* = 14) in the on-pump group and 1.6 % (*n* = 18) in the off-pump group (*P* = 0.347). The incidence of in-hospital mortality, in the patients who had a GI complication, was 28.6 % (*n* = 4) and 22.2 % (*n* = 4), respectively (*P* = 0.681).

Finally, a small-scale prospective study [35] examined gastric mucosal oxygenation together with whole-body oxygen flux in low-risk patients undergoing coronary artery bypass grafting with and without CPB. The authors concluded that despite superior global oxygen flux associated with beating-heart revascularization, gastric mucosal hypoxia occurred to similar extents in both groups with worsening trends for the OPCAB patients postoperatively.

### Neurocognitive outcome

With ageing population more frequently been referred for CABG, changes in neurocognitive function are more common in the postoperative setting and thus provide greater power for demonstrating improvement with changes in surgical technique.

Is the number of high intensity transient signals (HITS) measured by transcranial Doppler (TCD) reduced with off pump? Does that translate into clinically meaningful outcome favoring off pump? Furthermore, does it appear to be a superior early neurocognitive outcome with off pump in high-risk patients? Unfortunately, the up to now studies have failed to suggest so; A meta-analysis looking into neurocognitive outcomes after on versus off pump CABG, incorporating 892 patients show no benefit between techniques [36]. Kozora et al. [37] reported on 1156 patients (581 on-pump, 575 off-pump) completed match-paired neuropsychological assessments at baseline and 1-year follow-up. Only 20 % of either group had mild impairment at baseline on three of the test scores, and less than 10 % had severe impairment on individual tests at either time. Few subjects in either group transitioned to clinically impaired levels at follow-up on individual tests.

A small-randomized control trial by Van Dijk et al. [38] showed that Cognitive decline occurred in 21 % in the off-pump group and 29 % in the on-pump group, postoperatively. At 12 months, cognitive decline occurred in 30.8 % in the off-pump group and 33.6 % in the on-pump group, so the small differences between the 2 groups had become negligible. The same group reported on the 5 year cognitive function [39]; 33.3 % in the off-pump group and 35.0 % in the on-pump group had cognitive decline, therefore avoiding CPB had no effect on 5-year cognitive outcomes. An interesting study [40] looked at embolic signals detected with bilateral trans-cranial Doppler ultrasonography of the middle cerebral artery. Neurocognitive tests were administered preoperatively, on discharge from hospital, at 6 weeks, and at 6 months after surgery. Median number of embolic signals was 1605 (751 to 2473) during on-pump and 9 (4 to 27) in off-pump CABG (*p* < 0.001). The authors concluded that embolic load was prominent during on pump surgery and this possibly explains why the neurocognitive function was better immediately following off-pump surgery. This difference however was equalized at 6 weeks and 6 months.

Finally to add to the controversy of the impact of off-pump in neurocognitive function was a report of a long term follow up [41] on the subject; Patients undergoing off-pump coronary artery bypass showed better attention, performing better at tracking and mentally manipulating information, demonstrated better cognitive reasoning and also showed a trend toward better verbal learning. The authors however admit that these differences were small and of uncertain clinical importance.

A potential neuroprotective effect of pulsatile flow during off-pump surgery together with less aortic manipulation, may explain the findings of an important meta-analysis by Altarabsheh et al. [42]. Sixteen retrospective studies (9744 On pump and 8566 Off pump patients) were included in the systematic review. Stroke rates were higher in the On pump cohort (RR, 0.65; 95 % CI, 0.49–0.87; *p* < 0.01)

Misfeld et al. [43] interestingly carried out a systematic review of all published evidence that compares neurologic complications after anaortic off-pump coronary artery bypass grafting versus that with aortic manipulation.

Eight observational studies reported neurologic complications in 5619 anaortic off-pump coronary artery bypass grafting cases and 5779 cases with aortic manipulation. Postsurgical neurologic complications were significantly lower in anaortic off-pump coronary artery bypass grafting cases (odds ratio, 0.46; 95 % confidence interval, 0.29–0.72; I(2) = 0.8 %; *P* = .0008).

In summary, the impact of off pump in neurocognitive function is still under dedate however what is becoming clear is thatavoidance of aortic manipulation during off-pump coronary artery bypass grafting decreases neurologic complications relative to standard technique in which the ascending aorta is manipulated. In patients at high risk for stroke or transient ischemic attack, we recommend aortic non-touch technique during off-pump coronary artery bypass grafting.

### Systemic inflammation

Inflammatory mediators are observed after surgical trauma and following ischemia/re-perfusion. Such activation is also modulated by genetic factors.

Does the available literature permit definitive conclusions to be made on the advantages of off-pump surgery with respect to the systemic inflammatory response? In a small prospective randomized trial Formica et al. [44] compared the levels of systemic inflammation on mini CPB versus off-pump; Cardiac release of interleukin-6, tumor necrosis factor-alpha, and blood lactate were not different in both groups. Release of troponin T was not significantly different in both groups. Levels of creatine kinase mass were statistically higher in the mini CPB group than in the off-pump group, but only at the end of the operation. Hemoglobin levels were significantly higher in the mini CPB group than in the off-pump group after 24 h.

An elegant study [45] on the subject, looked at Plasma levels of C3bc, the terminal SC5b-9 complement complex, myeloperoxidase, beta-thromboglobulin and prothrombin fragment F1 + 2 before the operation, intra-operatively, at termination of the operation, and two hours post-operatively. 22 patients randomized to on-pump heart surgery and 22 to off-pump surgery. The authors found that off-pump surgery completely eliminated the heart-lung machine-induced complement activation. Neutrophils and platelets were equally activated in both groups as indicated by an early increase in myeloperoxidase and an increase in beta-thromboglobulin and F1 + 2, respectively, whereas interestingly, coagulation was enhanced post-operatively in the off-pump group.

Franke et al. [46] looked at the serum levels of pro-inflammatory interleukin (IL)-6, IL-8, tumor necrosis factor (TNF)-alpha as well as C-reactive protein (CRP), lipoprotein-binding protein (LBP) and procalcitonin (PCT) were measured up to the 5^th^ post-operative day in 108 patients divided into 3 groups: CPB, off pump and thoracic surgery. There was a relationship between IL-6 synthesis and the degree of surgical trauma. IL-8 was only elevated after cardiac surgery whereas pro-calcitonone liberation depended on the use of CPB. Trauma and reperfusion injury appeared to be more important contributors to acute inflammatory response compare to CPB. Vallely et al. [47] investigated endothelial activation (by measuring endothelial cell adhesion molecules) following on or off-pump; E-selectin, ICAM-1 and VCAM-1 expression into plasma were elevated 24 h post-operatively in both groups (*P* < 0.01), with no differences between the groups. Twenty-four hours post off-pump surgery plasma increased basal and IL-1beta induced expression of endothelial VCAM-1 by 133+/−16 % and 140+/−27 % (*P* < 0.05), respectively. Plasma taken 3 h post on pump CABG decreased endothelial VCAM-1 expression by 76+/−10 % (*P* < 0.05). Peri-operative plasma had no effect on endothelial expression of E-selectin or ICAM-1 in either group. Therefore off and on-pump appear to generate qualitatively different inflammatory responses with respect to endothelial activation.

In summary, although it seems that systemic inflammation is attenuated during off-pump surgery, the relationship between high levels of inflammatory mediators and clinical outcomes needs to be assessed in large patient populations to demonstrate to what extent off-pump surgery is more than just theoretically superior to on-pump surgery.

### Practical end-points, following off pump surgery

Limitation associated with the off pump technique, namely hemodynamic instability, concerns about the quality of the anastomosis, the ability to achieve complete revascularization and the on pump conversion rate, has raised concerns amongst the surgical community.

1. **Quality of the anastomosis**

Inferior results with off-pump has been attributed to surgeons lack of experience: in some trials the surgeons involved has performed less than 100 cases of which less than 15 % of coronary work had been performed off-pump. A surgeon volume-outcome relationship exists for mortality after off-pump with a threshold of more than 50 operations per year [48].

How does the quality of the anastomosis is compared between the two techniques? Khan et al. [49], showed significantly higher patency (97 versus. 89 %) after on pump versus off pump at 3 months post-operatively. However, Al-Ruzzeh et al. [50] looked at a randomized population of 168 patients were the graft patency was evaluated by angiography at 3 months; the authors reported similar patency between the on-pump and off-pump groups.

A very interesting large non-randomized trial, was reported at the STS meeting, by Puskas et al. [51]. The authors used the power of the STS database to provide direct comparison between on-pump and off- pump surgery. This resulted in a study group of 186,458 patients. Of that cohort, 65,864 underwent off-pump, whereas 120,594 underwent on-pump surgery. The off-pump group was more likely to be elderly people, women, and have suffered a preoperative stroke or renal failure, whereas the on-pump group were more likely to be diabetic, have a low ejection fraction congestive heart failure, more single and 2 vessel disease (34 % versus 19.6 %, *P* = <0.001), therefore requiring fewer grafts (3.04 versus 3.58, *P* = 0.001). The authors show a significant reduction in operative mortality in the off-pump group, as well as a highly significant reduction in overall adverse cardiac events, permanent stroke, dialysis, re-operation, prolonged ventilation, sternal wound infection, renal failure, and prolonged length of stay.

2) **Completeness of revascularization & Graft patency**

Completeness of revascularization with off pump techniques has been questioned and thus higher re-intervention rate has been reported in various studies.

Robertson et al. [52] studied whether patients undergoing off pump surgery are incompletely revascularized and whether this affects long-term survival and freedom from cardiac events.

Interestingly, on average, the patients undergoing Off pump received significantly fewer distal anastomoses than did those undergoing on pump (mean ± standard deviation, 2.6 ± 0.9 versus 3.0 ± 1.0, *P* < .0001). The circumflex territory was the most likely territory to be ungrafted during off pump in patients with angiographically significant obstruction (*P* = .0006). The frequency of complete revascularization was significantly different between the 2 groups (Off pump, 79.2 % versus on pump, 88.3 %; *P* = .0.002). The Off pump group had a significantly greater rate of total arterial grafting (Off pump, 66.6 % vs on pump, 49.7 %; *P* = .0001). No difference was seen in 8-year survival or freedom from cardiac cause hospital readmission between the 2 groups.

Moller et al. [53] reported on a meta-analysis of 66 randomized trials including 5202 patients. There were no statistically significant differences between the two techniques regarding mortality [relative risk (RR) 0.98; 95 % confidence interval (CI) 0.66–1.44], myocardial infarction (RR 0.95; 95 % CI 0.65–1.37), or repeat coronary revascularization (RR 1.34; 95 % CI 0.83–2.18).

Puskas et al. [54] randomized to off and on pump, 200 unselected patients (reoperations, patients in cardiogenic shock or patients who had preoperative IABP insertion were excluded). Complete revascularization was achieved in both groups.

Coronary angiography prior to hospital discharge on 93.4 % of enrolled patients was carried out and showed similar patency (99.0 % off-pump v. 97.7 % on-pump). Angiography at 1-year [55] in153 patients showed similar patency (93.6 % OPCAB v. 95.8 % ONCAB).

Likewise, Muneretto et al. [56] demonstrated that the 1-year angiographic patency was comparable between the 2 techniques.

Finally, follow-up angiograms in 1371 patients from the controversial ROOBY trial [57] who underwent 4093 grafts revealed that the overall rate of graft patency was lower in the off-pump group than in the on-pump group (82.6 % vs. 87.8 %, *P* < 0.01).

The primary ROOBY trial endpoints were a short-term composite (30-day operative death or major complications) and a 1-year composite (death, nonfatal acute myocardial infarction, or repeat revascularization). Secondary ROOBY endpoints included 1-year all-cause death, 1-year graft patency, 1-year changes from baseline in neurocognitive status and health-related quality of life, and costs.

The same authors reported that [58] for diabetic patients, the primary short-term composite outcome rate showed a worse trend for off-pump (8.0 %) than on-pump (3.9 %, *p* = 0.013), with no difference in the 1-year primary composite outcome or 1-year death rate. One-year patency was 83.1 % off-pump versus 88.4 % on-pump (*p* = 0.004). No differences were found in neurocognitive, health-related quality of life, discharge cost, and 1-year cumulative cost.

3) **Outcome results**

Off-pump coronary artery bypass grafting compared with coronary revascularization with the use of cardiopulmonary bypass is safe with comparable outcome in low-risk patients.

As per Lamy et al. [59] there is no significant difference between off-pump and on-pump CABG with respect to the 30-day rate of death, myocardial infarction, stroke, or renal failure requiring dialysis. The use of off-pump CABG resulted in reduced rates of transfusion, reoperation for perioperative bleeding, respiratory complications, and acute kidney injury but also resulted in an increased risk of early revascularization.

Moller et al. [60] randomly assigned 341 patients with a EuroSCORE > or = 5 and 3-vessel coronary disease to undergo coronary artery bypass grafting without or with cardiopulmonary bypass. The objective was to compare 30-day outcomes in high-risk patients randomized to on or off pump surgery. The primary outcome was a composite of adverse cardiac and cerebrovascular events (ie, all-cause mortality, acute myocardial infarction, cardiac arrest with successful resuscitation, low cardiac output syndrome/cardiogenic shock, stroke, and coronary reintervention). No significant differences in the composite primary outcome (15 % versus 17 %; *P* = 0.48) or the individual components were found at 30-day follow-up.

The criticism on this paper as per Pocar et al. [61] resides on the valuable comment that the Mean additive EuroSCORE was 6.9, but no patient with ejection fraction <30 % was enrolled, suggesting the prevalence of extracardiac risk factors in determining a higher risk profile.

The long-term results between the two techniques are comparable:

The long-term patency was studied with Coronary multi-sliced CT [62]. The likelihood of graft occlusion was no different between off-pump coronary artery bypass (10.6 %) and coronary artery bypass grafting with cardiopulmonary bypass (11.0 %) groups (odds ratio, 1.00; 95 % confidence interval, 0.55–1.81; *P* >0.99).

Furthermore a late follow up of a prospective randomized comparison of two hundred unselected patients undergoing off-pump versus conventional coronary artery bypass grafting by Puskas et al. [63], 190 grafts assessed by computed tomographic angiography, with comparable patency.

Wu et al. [64] looked at the state of New York’s Cardiac Surgery Reporting System and identify 2640 off-pump versus 5940 on-pump patients. The authors did a 1:1 propensity-score match 2631 patients and follow-up for a period of 7.2 years. There was no significant difference in long-term mortality (The 7-year survival rates were around 72 %) either overall or in their subgroup analysis of patients treated by high-volume surgeons who performed CABG in >50 % of their cases.

Long term experience with 1000 off pump cases, were reported by El-Hamamsy et al. [65]. Overall survival at 96 months was 74+/−3.5 % and cardiac survival was excellent at 94+/−1.3 %.

An interesting study by Kim et al. [66] evaluated long-term survival data in 5203 patients who underwent elective isolated CABG (off-pump, *n* = 2333; on-pump, *n* = 2870). Survival data were complete in 5167 patients (99.3 %) with a median follow-up duration of 6.4 years.

Both groups of patients showed a similar risk of death at 30-day (odds ratio, 0.70; 95 % CI, 0.35-1.40; *P* = 0.31) and up to one year (HR, 1.11; 95 % CI, 0.74-1.65; *P* = 0.62). For overall mortality, however, patients undergoing off-pump CABG were at a significantly higher risk of death (HR, 1.43; 95 % CI 1.19-1.71; *P* < 0.0001) compared with those undergoing on-pump CABG. Furthermore, long-term survival was similar between off-pump and on-pump CABG in patients undergoing non-emergent primary isolated CABG in Sweden from 1998 to 2008, as per Dalen et al. [67].

Hueb et al. [68] prospectively compared 155 off-pump patients with 153 on-pump patients who met their inclusion/exclusion criteria for their RCT. Follow-up at 5 years revealed no differences in composite end-points including death, MI, need for revascularization, angina recurrence or stroke.

Chaudhry et al. [69] sought to identify whether off-pump CABG conferred mortality or morbidity benefit over on-pump CABG in the long-term. Two of the 16 studies showed improved survival following on-pump surgery, whereas the remaining 14 showed no difference in late mortality (≥5 years). No differences were observed for other morbidity outcomes such as MACEs (including MI, recurrence of angina and heart failure or revascularization), stroke, graft patency, cognitive and quality of life.

A very important meta analysis of RCT was reported by Deppe et al. [70]. A total of 16 904 patients from 51 studies were identified. The incidence of MACCE did not differ between the groups, neither during the first 30 days nor for the longest available follow-up While the incidence of mid-term graft failure and the need for repeat revascularization was increased after off-pump surgery, on-pump surgery was associated with an increased occurrence of stroke renal impairment and mediastinitis. There was no difference with regard to hard clinical end-points between on- or off-pump surgery, including myocardial infarction or mortality.

Lastly, Moller et al. [71] selected to look at randomized clinical trials of off-pump versus on-pump CABG irrespective of language, publication status and blinding. The authors set off to assess the benefits and harms of off-pump versus on-pump CABG in patients with ischaemic heart disease.

The conclusions were quite striking; this analysis did not demonstrate any significant benefit of off-pump compared with on-pump CABG regarding mortality, stroke, or myocardial infarction. In contrast, the authors observed better long-term survival in the group of patients undergoing on-pump CABG with the use of cardiopulmonary bypass and cardioplegic arrest.

In summary both off- and on-pump surgery provide excellent and comparable results in patients requiring surgical revascularization. The choice for either strategy should take into account the individual patient profile (comorbidities, life expectancy, etc.) and importantly, the surgeon’s experience in performing on- or off-pump CABG in their routine practice.

4) **Re-intervention**

Recently, the results of the Coronary Artery Bypass Surgery Off or On Pump Revascularization Study (CORONARY) [72] has been published. This prospective study involved 4752 patients randomized to either on- or off-pump CABG in 79 centres and 19 countries. The use of off-pump CABG, as compared with on-pump CABG, did not reduce the rate of non-fatal stroke (1.0 % vs 1.1 %, respectively; *P* = 0.89) at 30 days or at one year (1.5 % vs 1.7 %, respectively; *P* = 0.24). Repeat revascularization rates at 1 year was similar between-group difference (3.1 versus 2.0 %; hazard ratio [HR], 1.52; 95 % confidence interval [CI], 0.90–2.54; *P* = 0.11)

Diegeler et al. [73] reported their results from the German Off-Pump Coronary Artery Bypass Grafts in Elderly Patients (GOPCABE) trial. This trial attempts to define the potential benefits of OPCAB in an elderly group (aged more than 75 years) with multiple comorbidities. A trend towards more 1-year repeat revascularization rates was observed in the off-pump group (1.4 versus 0.8 %; HR, 1.66; 95 % CI, 0.95–2.89; *P* = 0.07).

Furthermore, a meta analysis by Tagaki et al. [74] suggest that off-pump CABG may increase repeat revascularization rates by 38 % over on-pump CABG

The Predicting Long-Term Outcomes After Isolated Coronary Artery Bypass Surgery (PRIORITY) project [75] was designed to evaluate the long-term outcomes of 2 large, prospective multicenter cohort studies on CABG. The study population consisted of 11,021 patients who underwent isolated CABG (27.2 % off-pump CABG). Off-pump CABG thus carried a 42 % higher risk for subsequent percutaneous coronary intervention than on-pump CABG.

The current knowledge on the outcomes of “on versus off” pump surgery is summarized in a paper by Luo et al. [76]. In a meta analysis of RCTs there were no significant difference in all the short-term outcomes (mortality, myocardial infarction, stroke, renal failure, revascularization) and some long-term outcomes (mortality, myocardial infarction; stroke) between off-pump and on-pump CABG. However, off-pump CABG had a significantly higher revascularization rate than on-pump CABG in long-term follow-up.

## Conclusions

Extensive literature had been published on the subject of off pump coronary artery bypass surgery.

In terms of specific organ injury, there seems to be less neurocognitive deterioration/stroke, especially in high-risk patients, when anaortic techniques are used. There may be a small difference in favor of off pump technique in terms of renal and pulmonary injury. This again may be more significant in higher risk patients. No significant difference between the two techniques has been reported, in terms of gut protection.

Intuitively, avoiding the use of artificial circulation has reduced the inflammatory response invoked on the patients and in turn the transfusion requirement. However, for this technique to be further popularized, a translational benefit from the biochemical to the clinical level needs to be elucidated. Therefore, with the lack of robustness in the current evidence, more questions had been raised and there is currently still a controversy in its effectiveness and indications. Finally at present the argument of on pump versus off pump coronary surgery maybe shifted to [77] techniques that minimize surgical risk and maximize late survival after coronary artery bypass grafting.
